# A Narrowing of the Phenotypic Diversity Range after Large Rearrangements of the Karyotype in Salmonidae: The Relationship between Saltational Genome Rearrangements and Gradual Adaptive Evolution

**DOI:** 10.3390/genes8110297

**Published:** 2017-10-27

**Authors:** Alexander A. Makhrov

**Affiliations:** 1 A.N. Severtsov Institute of Ecology and Evolution of Russian Academy of Sciences, Moscow 119071, Russia; makhrov12@mail.ru; 2 Institute of Biophysics of Siberian Branch of Federal Research Center “Krasnoyarsk Science Center” of Russian Academy of Sciences, Akademgorodok, 50/50, Krasnoyarsk 660036, Russia

**Keywords:** evolution, karyotype, genome, morphology, ecology

## Abstract

The problem of how a gradual development of ecological and morphological adaptations combines with large genome rearrangements, which have been found to occur in the phylogeny of many groups of organisms, is a matter of discussion in the literature. The objective of this work was to study the problem with the example of salmonids, whose evolution included at least six events of multiple chromosome fusions. Large karyotype rearrangements are associated with a decrease in ecological and morphological diversity in salmonids. In the above example, genome rearrangements seem to distort the function of the genetic systems that are responsible for the occurrence of certain ecological forms in salmonids.

## 1. Introduction

During recent years, studies of evolutionary developmental morphology (MorphoEvoDevo), genetics and ecology have converged in the holistic approach [1,2,3]. However, there are still some unresolved problems in the field of research.

Particularly, large genome rearrangements unrelated to distant hybridization have been found to occur in many groups of organisms during their phylogeny. The set includes autopolyploidy [4], simultaneous multiple chromosome fusions [5], and mass transpositions of mobile genetic elements [6]. The findings are in conflict with the conventional Darwinian views of gradual adaptive evolution, as has been noted in several monographs [7,8,9,10]. It is clear that large genome rearrangements would dramatically change the genetic systems formed in earlier evolution, and thus distort respective adaptations in many cases.

How are large genome rearrangements compatible with a gradual development of adaptations? The problem is extremely difficult to solve, because morphological or ecological features of a group originating via a genome rearrangement are not necessarily a consequence of the rearrangement. To understand how morphological and ecological traits change as a result of large genome rearrangements, it is necessary to study several cases where rearrangements of the same type occur in related organisms. 

Such studies are possible, in particular, for autopolyploidy, which has occurred many times in evolution. An analysis of its evolutionary consequences in plants [11] and teleosts [12] has led to the idea of autopolyploidy as a nearly neutral process. Experimental data available for salmonids agree with the idea, apart from sterility in triploids [13,14]. However, functional divergence of many duplicated genes as a result of long−term evolution has been observed in polyploid groups, including those of salmonids [15,16,17]. 

Other chromosome rearrangements are usually deleterious for their carriers (for a review, see [18]). It is therefore difficult to search for neutral or adaptively advantageous chromosome rearrangements in an experimental setting. Yet such rearrangements, including large ones, have certainly occurred in evolution. 

For instance, simultaneous multiple chromosome fusions have repeatedly taken place in several groups of organisms [5]. Several such events have occurred in the evolution of salmonids. The group has two main karyotype categories, type A of karyotypes with approximately 80 chromosomes and type B of karyotypes with approximately 60 chromosomes [19]. There have been at least six events where a type B karyotype originated from a type A karyotype in the evolution of salmonids [20,21]. 

Balon [22] has associated a decrease in chromosome number with specialization in salmonids. A similar assessment was proposed by Qumsiyeh [23] with mammals. However, Pavlov and Osinov [24] have criticized the assumptions of Balon [22] in their comprehensive study. 

Based on published data, it is discussed belowwhether large karyotype rearrangements occurring independently in different phylogenetic lineages of salmonids have similarly affected their morphological and ecological diversity in a regular manner. Salmonids live in a variety of habitats, from small creeks to oceans, and are known to include a variety of ecological forms. However, the number of ecological forms differs among different salmonid species (Table 1). The species additionally vary in their ability to survive after spawning. The ability to colonize and to adapt to new habitats is another important, though rarely evaluated, sign of their phenotypic plasticity.

## 2. Atlantic Salmon *Salmo salar* L.

Atlantic salmon spawns in rivers of northeastern regions of North America and western and northern regions of Europe [29]. The species has 2n = 54–60, NF (chromosome arms) = 72–74 (for a review, see [26]). Atlantic salmon originated from an ancestor similar to the modern brown trout *Salmo trutta* on evidence of integrated data (for a review, see [30]). *Salmo trutta* has 2n = 76–84, NF = 92–104 (for a review, see [27]). Thus, fusions of several chromosomes accompanied the origin of Atlantic salmon [21,31].

Interestingly, the species *S. trutta* includes forms that have larger spawners and are similar in many ecological and morphological traits to Atlantic salmon, differing from the relatively small typical brown trout [30]. The most brilliant example is provided by Black Sea and Caspian Sea populations. The forms have been assigned to *Salmo salar* in several studies. Yet a karyotype analysis has allowed their exact species identification [30]. 

While substantial ecological diversity is characteristic of *S. trutta*, the majority of *S. salar* populations include only large individuals, which belong to the anadromous form, and dwarf males. Only a few populations include resident females, which are usually small in size [32]. The low frequency of these populations has been explained by the experimental finding that the early death rate in progeny of small−sized females is far greater than that of large−sized ones in *S. salar* ([33], and references therein).

Lower ecological plasticity of *S. salar* is seen well in South America, where the species has been introduced together with *S. trutta*. The latter has spread widely in the region, while *S. salar* is restricted to few large water systems [34].

As in all salmonids, *S. salar* is highly diverse, morphologically [35]. However, morphological diversity in *S. salar* is substantially lower than in the ancestral species *S. trutta* [32].

Thus, both large reorganization of the karyotype and a dramatic decrease in ecological and morphological diversity has occurred in *S. salar* during speciation. The karyotype reorganization might have considerably distorted the ability of small−sized freshwater females to mature, which was high in the ancestral species *S. trutta*. As is shown below, the hypothesis is supported by the fact that large karyotype rearrangements are associated with a decrease in ecological and morphological diversity in other salmonids. 

## 3. Pacific Salmonids (*Oncorhynchus*)

The number of species in the genus has been the matter of long−standing discussion. There are six species in the group (Table 2), in our opinion, which has been grounded previously [27]. All species of the genus *Oncorhynchus* spawn in rivers discharging into the Northern Pacific Ocean and the adjacent part of Arctic ocean [36]. 

A low chromosome number is characteristic of the majority of *Oncorhynchus* species (Table 1), which thereby belong to type B, according to the above classification [19]. The chum salmon *O. keta* is the only exception, occupying an intermediate place between types A and B. 

Females of the genus *Oncorhynchus* (with the exception of the cherry salmon *Oncorhynchus masou*) are single−time spawners (Table 2), while other salmonids (*Salmo*, *Parasalmo*, *Salvelinus*, *Parahucho*, *Hucho*, *Brachymystax*) are able to spawn more than once [37]. Of the 14 life−history strategies known for salmonids, only five have been observed in *Oncorhynchus* [25]. The capability of acclimatization is poor in salmons of the genus *Oncorhynchus* as compared with other salmonids [36]. Subspecies have not been recognized in the majority of the *Oncorhynchus* species, *O. masou* being the only exception [40].

Thus, a combination of a relatively low chromosome number with a decrease in ecological and morphological intraspecific diversity is characteristic of *Oncorhynchus* species. 

## 4. Pacific Trouts (*Parasalmo*)

*Parasalmo* found in tributaries of the North Pacific, and is commonly included in the genus *Oncorhynchus*. *Parasalmo* species have relatively low chromosome numbers, 56–68, with NF = 104–106 (for a review, see [19,27,28]. However, there are no signs of a decrease in morphological and ecological diversity in the group. In particular, the rainbow trout *Parasalmo mykiss* is one of the most plastic salmonid species, has a broad natural geographic range in the North Pacific (monograph: [41]), and has successfully been introduced into many rivers and lakes in various regions of the globe [36]. 

According to Balon [22], pedomorphosis accompanied the origin of *Parasalmo* and led to despecialization in this evolutionary branch. Whichever the case, large chromosome rearrangements do not always restrict morphological and ecological diversity in salmonids. 

## 5. Long−Finned Charr *Salvethymus svetovidovi*

This species is found only in the El’gygytgyn Lake of the Chukchi Peninsula (Russia), and is the only species of the genus *Salvethymus* [42], although the independent status of this genus is strongly doubted now [43,44]. The *Salvethymus svetovidovi* karyotype consists of 56 chromosomes with NF = 98 [28]. Arctic charrs of the genus *Salvelinus*, which are the closest relatives to *S. svetovidovi*, have 76–86 chromosomes with NF = 98–100 [28]. Thus, substantial karyotype changes in the form of multiple chromosome fusions accompanied the origin of *S. svetovidovi*.

It is important to note that some morphological features of *S. svetovidovi* (a great number of gill rakers and reduction of certain bones) are still expressed, but to a lesser extent in char forms from Trans−Baikal lakes [45]. Like in the case of *S. salar*, a large karyotype rearrangement occurring in the course of speciation might have allowed *S. svetovidovi* to preserve only part of the morphological diversity spectrum characteristic of its ancestor belonging to the genus *Salvelinus*. Morphological and ecological diversity is extremely high in *Salvelinus* (Table 1; for a review, see [46,47,48,49]).

The species *S. svetovidovi* includes only one population, and has never been the subject of experimental research [50]; the stability of its morphological and ecological traits is therefore difficult to estimate. Yet the form seems to be conserved. The form diverged to a great extent from two sympatric forms of the genus *Salvelinus*, and its divergence was allopatric, as molecular genetic studies have shown [44]. It is noteworthy that the two forms have somehow found their way into the El’gygytgyn Lake, while *S. svetovidovi* has not spread to other lakes. 

## 6. Sakhalin Taimen *Parahucho perryi*

The Sakhalin taimen karyotype includes 62 chromosomes with NF = 100 [19,28]. Different genera have been identified as sister groups of *Parahucho* by different molecular genetic methods, the set including *Hucho* [51,52], *Salmo* [53,54], and *Salvelinus* [52,53,55]. The chromosome number is far greater, approximately 80, in all of these groups (for a review, see [19,28]. Thus, multiple chromosome fusions occurred during the formation of *Parahucho perryi*, which is the only species in the genus *Parahucho*.

The *P. perryi* species range is relatively small; spawning occurs in rivers of Sakhalin, the Kuril Islands, Hokkaido, and the Russian coast of the Sea of Japan [56]. Gritsenko [56] have summarized the data available for *P. perryi* and characterized the species as a phenotypically homogeneous, monotypic species. 

Ecological plasticity is also limited in *P. perryi* [56]. Distinct ecological forms are apparently lacking. The species uses river, lake, and estuarine waters for feeding [57,58,59], although capable of living in marine waters [60]. Dwarf spawners have not been mentioned for the species in the available literature. 

Thus, a combination of a dramatic decrease in chromosome number with a relatively low morphological and ecological diversity is observed again in *P. perryi*.

## 7. Broad Whitefish *Coregonus nasus*

The diploid chromosome set includes approximately 80 chromosomes in the majority of coregonids, while the broad whitefish *Coregonus nasus* has 2n = 58–60, NF = 92–98 (for a review, see [19,28]). 

In a monograph focusing on coregonid fishes, Reshetnikov ([61], p. 170) has characterized *C. nasus* as a relatively stable species wherein subspecies or geographical forms can hardly be isolated objectively. Broad whitefish usually lives in rivers and is not found in waters with a salinity higher than 9–15‰ [61]. 

*Coregonus lavaretus* and *C. clupeaformis*, which are the closest relatives to *C. nasus* [62], have very high morphological and ecological diversity ([61,63,64,65]. In particular, either species forms morphologically different populations in different geographic regions, has anadromous forms, and quickly adapt to new habitats (Volga tributaries, the Lake Sevan in the Caucasus, etc.). 

Thus, a relatively low chromosome number and relatively low phenotypic diversity are characteristic of *C. nasus*.

## 8. Immobilization: An Association of Gradual and Saltational Evolution

It is important to note that a decrease in ecological and phenotypic diversity is restricted to the widespread salmonid species and genera that have a lower chromosome number (Table 1). The fact strongly suggests that chromosome fusions can explain why phenotypic diversity declines over time in some species.

Species with a low morphological and ecological plasticity thus originated from species that included many ecological forms, with each form having its morphological specifics. Note that the phenomenon differs from specialization, which is adaptation to particular way of life. The phenomenon is not adaptive; it is known as immobilization, that is, a reduction of evolutionary plasticity [66]. Unfortunately, this interesting phenomenon has only recently come to be studied in detail [67].

It is important to understand that immobilization does not mean that the species becomes totally incapable of adaptation as a result. For example, pink salmon has almost lost the capability to form resident populations, but is still highly competitive in a marine environment. 

Gradual evolution was apparently combined with saltational evolution in salmonids. First, numerous forms that differ in ecology and morphology arise via gradual adaptive evolution within an ancestral taxon. Then, the capability of producing such forms is lost, potentially as a result of chromosome fusions in some species. 

Thus, gradual evolution is not necessarily an alternative to saltational evolution, but the two respective scenarios may complement each other. A new species undergoing a large genome rearrangement will, in fact, hardly survive if expressing absolutely new phenotypic characters, which are extremely unlikely to be adaptive. Yet the species may survive if expressing the ecological and morphological characters that arose in the ancestral lineage via gradual adaptive evolution; these characteristics may currently be seen in particular groups within the related species. 

Cases of rather large chromosome rearrangements are considered here, but other changes of the genome are probably capable of immobilizing ecological and morphological characters as well. For instance, a deletion or loss of expression of a gene can eliminate the pathways that lead to alternative phenotypes [68,69].

The genome is a complex system that includes many regulatory elements [70]. A large genome rearrangement is therefore unlikely to immediately result in a new adaptation (although they open new opportunities for adaptations to arise). Yet such a rearrangement can easily and immediately eliminate one of several discrete adaptive norms (alternative adaptive phenotypes).

In the above example, genome rearrangements seem to distort the function of the genetic systems that are responsible for the occurrence of certain ecological forms in salmonids. For instance, resident forms can hardly develop in Atlantic salmon. Dysfunction of genetic systems is not an obligatory consequence of genome rearrangements; e.g., no sign of a decrease in morphological and ecological plasticity is seen in *Parasalmo*.

Immobilization, described above, yields organisms that display robustness; that is, lack of phenotypic variation in several characters. This robustness may favor adaptation in certain cases [71].

## 9. Conclusions

The examples known in salmonids demonstrate that large karyotype rearrangements are may be capable of reducing ecological and morphological diversity. It is clear from the above that gradual (adaptive) and saltational (nonadaptive) modes of evolution are not necessarily alternative. A plausible evolutionary scenario is that adaptations arise in the course of gradual evolution and are then fixed as a result of a genome rearrangement. The exact molecular mechanisms underlying the saltational evolution described here related to chromosome fusions is yet to be determined, and will be an important avenue of investigation for future studies.

## Figures and Tables

**Table 1:** Types of anadromous and resident life strategy in Salmoninae ([25], with addition) and their chromosome number (2n) [19,26,27,28].

Species	Types of Strategy *														2n
	1	2	3	4	5	6	7	8	9	10	11	12	13	14	
*Salmo salar*	+	−	?	+	+	+	−	−	−	−	−	−	+	+	54–60
*Salmo trutta*	+	+	+	+	+	+	−	+	+	+	+	−	+	+	76–84
*Oncorhynchus gorbusha*	+	−	−	−	−	−	−	−	−	−	−	−	−	−	52–54
*Oncorhynchus keta*	+	−	−	−	−	−	−	−	−	−	−	−	−	−	74
*Oncorhynchus nerka*	+	−	−	−	−	−	−	−	−	−	−	+	+	−	57,58
*Oncorhynchus kisutch*	+	−	+	−	+	−	−	−	−	−	−	−	+	−	60
*Oncorhynchus tschawytcha*	+	−	+	−	+	−	−	−	−	−	−	−	−	−	68
*Oncorhynchus masou*	+	−	+	−	+	+	−	−	−	−	−	−	+	−	66
*Parasalmo mykiss*	+	+	+	+	+	−	−	+	+	+	+	−	+	+	58–65
*Parasalmo clarki*	?	+	−	+	+	+	−	+	−	+	?	−	+	+	64–68
*Salvethymus svetovidovi*	−	−	−	−	−	−	−	−	−	−	−	−	+	−	56
*Salvelinus alpinus/malma* complex	?	−	?	−	+	+	+	+	+	+	+	−	+	+	76–82
*Parahucho perryi*	+	−	−	−	−	−	−	+	−	+	−	−	−	+	62

* (1) Typically migratory (2) smolts initially feed in seawater, return for wintering to rivers, and only then finally go for feeding to the sea until maturation (3) jacks, (4) half−pounders (thousanders, postsmolt, pregrilse), (5) dwarf riverine males, (6) dwarf riverine females, (7) facultatively anadromous, (8) estuarine, (9) riverine–estuarine, (10) riverine, (11) resident males and females which having spawned in fresh water may make downstream migration to the sea and return already as migratory fish, (12) resident dwarf lacustrine *Oncorhynchus nerka* (predominantly males as a part of the stock of migratory form), (13) lacustrine, (14) lacustrine–riverine, ? – no precise data.

**Table 2:** Characteristics of species of the genus *Oncorhynchus.*

Species	Dwarf River Females and Males ([25], with Addition)	Multiple Spawning ([37], with Addition)
Pink salmon *O. gorbuscha*	No	No
Chum salmon *O. keta*	No	No
Sockeye salmon *O. nerka*	No	Possible only in resident males. However, females that survive spawning are found in some lakes where resident *O. nerka* has been introduced [38]
Coho salmon *O. kisutch*	Dwarf males present	Possible only in resident males
Chinook salmon *O. tshawytscha*	Dwarf males present	Possible only in dwarf males
Cherry salmon *O. masou*	Dwarf males present; “female parr maturation requires extremely favorable growth conditions” ([39], p. 962)	Multiple spawning present
